# Generating datasets for the project portfolio selection and scheduling problem

**DOI:** 10.1016/j.dib.2022.108208

**Published:** 2022-04-25

**Authors:** Kyle Robert Harrison, Saber M. Elsayed, Ivan L. Garanovich, Terence Weir, Sharon G. Boswell, Ruhul A. Sarker

**Affiliations:** a School of Engineering and Information Technology, University of New South Wales, Canberra ACT 2600, Australia; b Defence Science and Technology Group, Department of Defence, Canberra ACT 2610, Australia

**Keywords:** Project portfolio selection, Project scheduling, Portfolio optimisation, Benchmark problems

## Abstract

The article presents two variants of the project portfolio selection and scheduling problem (PPSSP). The primary objective of the PPSSP is to maximise the total portfolio value through the selection and scheduling of a subset of projects subject to various operational constraints. This article describes two recently-proposed, generalised models of the PPSSP [1], [2] and proposes a set of synthetically generated problem instances for each. These datasets can be used by researchers to compare the performance of heuristic and meta-heuristic solution strategies. In addition, the Python program used to generate the problem instances is supplied, allowing researchers to generate new problem instances.

## Specifications Table

**Table:** 

Subject	Management Science and Operations Research
Specific subject area	Project portfolio selection and scheduling
Type of data	JSON files
	Python program
How data were acquired	Artificially generated by a Python 3.9 program, which is included in the dataset
Data format	Raw
	Software
Description of data collection	All data has been synthetically generated using Python
Data source location	Institution: School of Engineering and Information Technology, University of New South Wales
	City/Town/Region: Canberra
	Country: Australia
Data accessibility	Repository name: Datasets for the Project Portfolio Selection and Scheduling Problem
	Data identification number (DOI): 10.17632/7v65s7k4y8.1
	Direct URL to data: https://data.mendeley.com/datasets/7v65s7k4y8/1

## Value of the Data

• This dataset provides a set of problem instances from two generalised PPSSP formulations that can be used by researchers to provide comparative analyses of new optimisation techniques.
• These problem instances will benefit researchers involved in the selection and scheduling of project portfolios.
• These problem instances allow for direct comparison of heuristic and meta-heuristic optimisation techniques, thereby providing a standard baseline for performance comparison.
• The Python program used to generate the problem instances is also provided, facilitating the generation of new problem instances, as required.

## 1 Data Description

This article describes two datasets, each containing a wide variety of benchmark problem instances that are generated according to one of two models. The supplied JSON files each consist of a single problem instance, as detailed below. The primary objective of each benchmark problem is to maximise the portfolio value resulting from the selection and scheduling of projects, subject to various operational constraints. However, the two models differ significantly in how the projects are selected for implementation and how the total portfolio value is calculated. The full model formulations are provided in Appendix A.

In the first model, extended from [1], the selection and valuation are both done at the project level. Table 1 describes the parameters used to generate the problem instances. In Table 1, the tuple (g,p) represents that group of size g was generated with probability p. Furthermore, the budget for each problem instance in the first model was set as $15n_{p}+n_{p}\left(t-1\right)$. That is, the initial budget was 15 times the number of projects and was increased by an amount equal to the number of projects for each year in the planning window. There are 2592 problem instances generated according to this model, which are supplied in the “project_instances” folder. The problem instances in this folder are divided into three sub-folders (250, 500, 1000) based on the number of projects contained in the problem. Within each sub-folder, instances are named “PI_{index}_{number of projects}_{number of planning years}_{proportion of budget for initiating projects}_{proportion of budget for maintaining ongoing projects}_{time discount rate}.json”. Each JSON file contains the following properties:

• problem_name (string) – the name of the problem instance.
• periods (integer) – the number of planning periods, in years.
• budget (list of real values) – the total available budget in each year.
• initiation_budget (list of real values) – the maximum budget for starting new projects in each year.
• maintenance_budget (list of real values) – the maximum budget for maintaining ongoing projects in each year.
• synergies (list of objects) – a list of synergy relationships, which are realised if all projects within the relationship are selected for implementation. Each synergy object has the following properties:
  – value (real value) – the value added if all listed projects are selected for implementation.
  – project_ids (list of integers) – a list of the project IDs associated with this synergy relationship.
• projects (list of objects) – a list of all projects contained in this problem instance. Each project object has the following properties:
  – id (integer) – a unique ID associated with this project.
  – name (string) – the project name.
  – duration (integer) – the duration of the project, in years.
  – prerequisites (list of integers) – the IDs of prerequisite projects that must be completed before this project can be initiated.
  – successors (list of integers) – the IDs of projects that can only be initiated if this project is completed.
  – mutual_exclusions (list of integers) – the IDs of projects that can not be selected for implementation if this project is selected.
  – cost (list of real values) – the yearly cost associated with implementation of this project.
  – value (list of real values) – the yearly value associated with implementation of this project, without time-discounting.

**Table 1:** Problem instance parameter values for the first, project-oriented model.

Parameter	Values
Projects	{250, 500, 1000}
Planning window (years)	{15, 20, 25}
Starting and ongoing budget limits	{None, (25%, 75%)}
Prerequisites	{None, [(2, 25%)], [(3, 25%)], [(2, 50%), (3, 50%)]}
Mutual exclusions	{None, [(2, 25%)], [(3, 25%)], [(2, 50%), (3, 50%)]}
Synergy groups	{None, [(3, 10%), (5, 10%)], [(3, 25%), (5, 25%)]}
Time discount rate	{0.00, 0.01, 0.03}

In the second model, originally proposed in [2], selection and valuation is done via predetermined groups of projects, referred to as options. Table 2 describes the parameters used to generate the problem instances. There are 768 problem instances generated according to this model, which are supplied in the “option_instances” folder. The problem instances in this folder are divided into four sub-folders (25, 50, 100, 250) based on the number of families contained in the problem. Within each sub-folder, instances are named “OI_{number of families}_{number of projects}_{proportion of divestment projects}_{yearly budget deviation}_{number of planning years}.json”. Each JSON file contains the following properties:

• budget_years (integer) – the number of budget planning periods, in years.
• yearly_allowance – the allowable yearly budget deviation.
• projects (list of objects) – a list of all projects contained in this problem instance. Each project object has the following properties:
  – name (string) – the project name.
  – preferred_start (integer) – the preferred (default) start time for this project.
  – earliest_start (integer) – the earliest allowable start time for this project.
  – latest_start (integer) – the latest allowable start time for this project.
  – effective_time (integer) – the number of time periods after initiation before this project begins delivering an initial value.
  – yearly_cost (list of real values) – the yearly cost associated with implementation of this project, which may be negative for divestment projects.
• families (list of list of objects) – the list of families in this problem instance, such that each family is a list of options. Each option has the following properties:
  – name (string) – the name of the option.
  – projects (list of string) – a list of project names associated with this option, which may be empty.
  – values (list of real values) – the yearly value associated with option. Note that, the value is note delivered over time. Rather, a single value is selected depending on the period in which all projects in this option have reached their effective time.

**Table 2:** Problem instance parameter values for the second, option-oriented model.

Parameter	Values
Families	{25, 50, 100, 250}
Projects	{100, 250, 500, 1000}
Divestments	{10%, 25%, 33%, 50%}
Allowable yearly budget deviation	{$0, $500, $1,000 }
Planning window	{10, 15, 20, 25}

Finally, this dataset contains the Python source code (“PPSSP_datagen-master”) that can be used to replicate the supplied problem instances or create new instances by independently varying any of the parameters listed in Tables 1 and 2, respectively. By default, the main script (“main.py”) will regenerate all problem instances supplied in this dataset, written in the current directory. However, the output directory can be configured via the “base_directory” variable.

## 2 Experimental Design, Materials and Methods

The process to generate the synthetic data follows statistical distributions that were derived to resemble the characteristics of the future force design problem encountered by the Australian Department of Defence. However, the models and datasets are generally applicable and not specific only to applications in the Defence sector. All problem instances, including the project data, were synthetically generated using a Python program, which is also supplied in this dataset. For each problem instance in the first problem set, a large set of representative projects was first generated. The data generation process was designed to generate project data that has similar characteristics to those found in the publicly-available 2016 Integrated Investment Plan, published by the Australian Department of Defence [3]. To generate synthetic data for each project, the duration and total cost were first sampled, independently for each project, from a multivariate log-normal distribution given by

(1) $$\left[\begin{array}{c} d_{p}\\ c_{p} \end{array}\right]∼\mathrm{lognormal}\left(\left[\begin{array}{c} 2.191054\\ 6.642006 \end{array}\right],\left[\begin{array}{cc} 0.246245 & 0.374572\\ 0.374572 & 1.555780 \end{array}\right]\right).$$

Both values were rounded to the nearest integer value. The multi-variate log-normal distribution generates cost profiles that have increasing expenditures up to a maximal cost then slowly decrease, which matches the expenditure profiles found in Defence projects [4]. Given that the Weibull distribution has been shown to accurately model cumulative expenditure profiles [4], [5], a normalised cumulative distribution function (CDF) of the Weibull distribution was used to distribute the total cost among each year of a project’s lifetime. The CDF was used to dictate the cumulative total percentage of a project’s expenditure based on the completion percentage at each time period. Specifically, the CDF describing the (cumulative) yearly expenditure profile of each project is given by

(2a) $$CDF_{c_{it}}=\frac{1-\exp{\left(\frac{t}{\beta }\right)}^{\alpha }}{1-\exp{\left(\frac{1}{\beta }\right)}^{\alpha }},\forall t\in 1,2,\ldots ,d_{p}$$

where

(2b) α∼N(1.589,2)

(2c) β=max[N(0.71,0.3),0.1].

Note that, if the value of α fell below 0.1, it was regenerated using Eq.  (2b). For each year in a project’s lifetime, the cost was taken as the difference in the cumulative proportion between the current year and that of the previous year, rounded to the nearest integer value.

The total value associated with a project is based on both its total cost and duration according to

(3) $$v_{p}=U\left(0,2\right)c_{p}+\sum_{j=2}^{d_{p}}∼U\left\{1,4\right\},$$

where U{1,4} generates a random integer between 1 and 4, both inclusive. In general, this methodology associates a higher total value with projects that either have a higher total cost or longer duration. A similar cost-value relationship was employed in Fleischer Fauske et al. [6]. Similar to how the cost is distributed over each period in the project’s lifetime, the yearly value was distributed over each period using the CDF given in Eq.  (2).

To generate the prerequisite constraints, a user-supplied proportion of projects were associated with precedence groups. For each precedence group, a number of projects were randomly selected without replacement.^1^ Without loss of generality, in each group of projects $\left(p_{g1},p_{g2},\ldots ,p_{gn}\right)$, with indices g1<g2<…<gn, it was assumed that $p_{g1}$ was a prerequisite for projects $\left(p_{g2},\ldots ,p_{gn}\right)$. Groups of projects that were mutually exclusive were generated in the same fashion, with the exception that all projects within each group were considered to be mutually exclusive. Synergy groups also followed the same generation process. To determine the value for a synergy group, a random value was selected between 1 and the (non-discounted) total value of all projects within the group.

For the second set of problem instances, the cost profile for each project is characterised by a randomly-determined Gaussian distribution. As with the first set of problem instances, the statistical distributions were designed to provide characteristics that are similar to those found in Defence projects. To generate the cost profile, the current year was first defined as y. Then, a mean year ($\mu_{y}$) was selected as a random integer between (y−5) and (y+5) and a standard deviation ($sd_{y}$) as selected as a random integer between 1 and 10. The project’s preferred start time was then taken randomly between the current year and the final budget period, while the end year is (nominally) taken as the year in which a project ceases its expenditure. For investment projects, the effective time ($te_{p}$) was determined as a random integer between 3 and the number of periods remaining in the planning period after the project’s default start time. Divestment projects were assumed to take effect immediately (i.e., $te_{p}=0$).

To generate the cost, a value m was defined as the ratio between a randomly generated scale (s∼U{1,10}) and randomness parameter (r∼U{100,s}), i.e., m=s/r. All costs prior to the start year were taken as zero. The cost was then generated such that it was increasing for each year (t) up the mean year $\mu_{y}$ according to

(4) $$CP_{pt}=\max\left(\text{PDF}\left(N(\mu_{y},sd_{y}),i\right)\times s+U\left(-m\ldots m\right),CP_{p(t-1)},0\right),$$

and decreased for each year after the mean year according to

(5) $$CP_{pt}=\max\left(\text{PDF}\left(N(\mu_{y},sd_{y}),i\right)\times s+U\left(-m\ldots m\right),CP_{p(t+1)},0\right).$$

where $N(\mu_{y},sd_{y})$ is a Gaussian distribution with mean $\mu_{y}$ and standard deviation $sd_{y}$ and PDF is the probability density function. Note that, the yearly costs after the mean year must be generated in reverse order given that they are bounded by the cost associated with the succeeding year in Eq.  (5). To provide the cost profile for divestment projects, the yearly costs were negated in 50% of the generated projects.

To associate projects with options, the projects were first split based on whether they were divestments or investments, i.e., whether their total net cost was less than or greater than zero. The number of options associated with each family was randomly chosen between 2 and 6, both inclusive. Then, a group of 6 projects was selected such that all projects were either investments or divestments. For each option in the family (except the first, “empty” option), a unique subset^2^ of these 6 projects was randomly chosen. Notably, these subsets could contain between 1 and 6 projects, thereby facilitating the generation of options with differing numbers of projects.

To assign a value profile to each option, an upper bound was randomly selected between 1.5 and 4. For each four-year window in the planning period, a value was randomly selected between 0 and the generated upper bound, then was assigned to each of the four years. Values were negated for options containing divestment projects. The generated list of values was then sorted, either descending for investment options or ascending for divestment options, such that earlier start times led to higher valuations for each option.

## CRediT authorship contribution statement

**Kyle Robert Harrison:** Writing – original draft, Software, Methodology. **Saber M. Elsayed:** Writing – review & editing, Funding acquisition. **Ivan L. Garanovich:** Writing – review & editing. **Terence Weir:** Writing – review & editing, Formal analysis. **Sharon G. Boswell:** Writing – review & editing, Project administration, Supervision. **Ruhul A. Sarker:** Writing – review & editing, Project administration, Funding acquisition, Supervision.

## Declaration of Competing Interest

The authors declare that they have no known competing financial interests or personal relationships that could have appeared to influence the work reported in this paper.

## Data Availability

Datasets for the Project Portfolio Selection and Scheduling Problem (Original data) (Mendeley Data).

## Appendix A Formulation of the Models

In this appendix, the mathematical formulations underlying the benchmark problem instances are provided.

### A1 Formulation of the first, project-oriented model

To describe the formulation underlying the first set of benchmark problem instances, the following notation is used:

• N is the total number of projects in the problem instance.
• T is the number of time periods considered for project initiation.
• $x_{it}$ is the (binary) decision variable. The value of $x_{it}$ is set to 1 in the time period t where project i is scheduled to be started; otherwise it is set to 0.
• $v_{it}$ is the value associated with project i in period t of its implementation. Note that, the value of t is relative to the time period in which project i is initiated.
• r is the time-discount factor for this problem instance.
• $c_{it}$ is the cost of project i in period t of its implementation.
• $B_{t}$ is the budget available to spend in period t.
• $IB_{t}$ is the initiation budget in period t. This is the amount of funds that can be allocated to projects that will begin in period t.
• $OB_{t}$ is the maintenance budget in year t. This is the amount of funds that can be allocated to continue projects that have started prior to time period t.
• $d_{p}$ is the duration of project p, in periods.
• $T_{B}$ is the number of periods considered for budget purposes and is defined as the number of planning periods (T) plus the maximum project duration ($\max(d_{p})$). This allows for budgetary considerations associated with projects continuing beyond the end of the planning period.
• PC(p) is a list of prerequisite projects that must be completed before project p can be initiated.
• ME(p) is a list of projects that are mutually exclusive to project p, such that only one from the set can be selected for implementation.
• SG is the set of synergy groups such that an extra value is realised if all projects from a synergy group are selected.
• $sv_{jk}$ is the value added if all projects in synergy group j have been started in period k.
• $g_{jk}$ is an ancillary variable that takes the value of 1 if synergy j is activated in period k; otherwise it is set to 0.

Firstly, the ancillary variables $g_{jk}$ are defined such that they take the value of 1 if all projects contained in synergy group j have been initiated in period k:

(A.1a) $$g_{jk}=\prod_{i\in SG_{j}}\sum_{t=\max(1,k-d_{i}+1)}^{k}x_{it},\;\forall j\in 1,2,\ldots ,\left|SG\right|,\forall k\in 1,2,\ldots ,T.$$

The model considered in the benchmark problem instances can then be formulated as:

(Maximise total portfolio value, including synergies, across all years):

(A.1b) $$\max\sum_{i=1}^{N}\sum_{k=1}^{T}\sum_{t=1}^{k}x_{it}\frac{v_{i,k-t+1}}{{\left(1+r\right)}^{k}}+\sum_{j=1}^{\left|SG\right|}g_{jk}sv_{jk}$$

subject to

(Yearly budget constraints):

(A.1c) $$\sum_{i=1}^{N}\sum_{t=1}^{k}x_{it}c_{i,k-t+1}\leq B_{k},\;\forall k\in T_{B}$$

(Initiation budget):

(A.1d) $$\sum_{i=1}^{N}\sum_{t=1}^{T_{B}}x_{it}c_{i,1}\leq IB_{k},\;\forall k\in T_{B}$$

(Maintenance budget):

(A.1e) $$\sum_{i=1}^{N}\sum_{t=1}^{k}x_{it}c_{i,k-t+1}\leq OB_{k},\;\forall k\in 2,3,\ldots ,T_{B}$$

(Precedence constraints):

(A.1f) $$\sum_{t=1}^{T}x_{jt}\geq \sum_{t=1}^{T}x_{it},\;\forall i\in N\;\text{and}\;\forall j\in PC\left(i\right)$$

(A.1g) $$\sum_{t=1}^{T}tx_{it}\geq \sum_{t=1}^{T}\left(t+d_{j}\right)x_{jt},\;\forall i\in N\;\text{and}\;\forall j\in PC\left(i\right)$$

(Mutual exclusion constraints):

(A.1h) $$\sum_{t=1}^{T}x_{it}+x_{jt}\leq 1,\;\forall i\in N\;\text{and}\;\forall j\in ME\left(i\right)$$

(Single selection):

(A.1i) $$\sum_{t=1}^{T}x_{it}\leq 1,\;\forall i\in N.$$

The objective function given in Eq.  (A.1b) calculates the total portfolio value of all projects. This includes both the time-discounted project value in each year, using a discount rate of r, and any synergy effects that are realised by Eq.  (A.1a). Time discounting is used as a mean to prioritise the earlier delivery of value in the planning period, such that the value of a project is decreased each period. Eq.  (A.1c) enforces the total budget is respected in all time periods by accounting for the costs of all projects currently implemented. Eqs.  (A.1d) and (A.1e) ensure that the budgets for starting new projects and maintaining ongoing projects, respectively, are adhered to. Eqs.  (A.1f) and (A.1g) ensure that all prerequisite projects, if applicable, have been completed before a project can be initiated. Eq.  (A.1h) ensures that only project from a mutual exclusion group, if applicable, is selected for implementation. Eq.  (A.1i) ensures that projects are not selected for implementation more than once.

### A2 Formulation of the second, option-oriented model

To describe the formulation underlying the second set of benchmark problem instances, the following notation is used:

• P is the set of projects.
• $O_{i}$ is the set of options associated with family i.
• $F_{ij}$ is the set of projects associated with option j in family i.
• I is the set of families.
• $y_{ij}$ is a (binary) variable that takes the value of 1 if option j is selected from family i; otherwise it is set to 0.
• $z_{pt}$ is a (binary) variable that takes the value of 1 if project p is scheduled to be initiated in time period t; otherwise it is set to 0.
• $d_{p}$ is the duration of project p (in years).
• $te_{p}$ is the effective time of project p, i.e., the number of periods before this project delivers an initial level of benefit.
• $EST_{p}$ is the earliest period in which project p can be initiated.
• $LST_{p}$ is the latest period in which project p can be initiated.
• $NP_{ij}$ is the number of projects associated with option j in family i.
• $CP_{pt}$ is cost associated with project p in period t.
• $BD_{t}$ is the amount of budget deviation that is allowable in time period t.
• $E_{ij}$ is the effective time of option j in family i, defined in Eq.  (A.2b).
• $VO_{ijt}$ is the value of option j in family i when its effective time is period t.

Firstly, to determine the set of projects that are active in time t, the variable $z_{pt}$ is introduced as:

(A.2a) $$\sum_{t=k-d_{p}+1}^{k}z_{pt}=1.$$

Then, to determine the effective time of an option, the earliest time period such that all included projects are able to provide their initial benefit must be determined. For each project, this is based on the period in which the project is started and its effective time ($te_{p}$). If an option is not selected, $E_{ij}$ will be 0; otherwise, the effective time for an option can be calculated as:

(A.2b) $$E_{ij}=\max_{p\in F_{ij}}\sum_{t=EST_{p}}^{LST_{p}}z_{pt}\left(t+te_{p}\right),\;\forall i,j.$$

Then, the formulation of this model can be given by:

(Maximise total portfolio value):

(A.2c) $$\max\sum_{i=1}^{\left|I\right|}\sum_{j\in O_{i}}y_{ij}\times VO_{ijE_{ij}}$$

subject to:

(Exactly one option must be selected from each family):

(A.2d) $$\sum_{j\in O_{i}}y_{ij}=1,\;\forall i\in I$$

(All projects within an option be implemented)

(A.2e) $$\sum_{p\in F_{ij}}\sum_{t=EST_{p}}^{LST_{p}}z_{pt}=NP_{ij}\times y_{ij},\;\forall i,j$$

(Projects must be initiated within their allowable window):

(A.2f) $$\sum_{t=EST_{p}}^{LST_{p}}z_{pt}\leq 1,\;\forall p\in P$$

(Yearly budget and allowable deviation):

(A.2g) $$\sum_{p=1}^{\left|P\right|}\sum_{t=k-d_{p}+1}^{k}z_{pt}\times CP_{pk}\leq BD_{k},\;\forall k$$

(Binary variable constraints):

(A.2h) $$y_{ij}\in \left\{0,1\right\},\;\forall i,j$$

(A.2i) $$z_{pt}\in \left\{0,1\right\},\;\forall p,t$$

The objective given in Eq.  (A.2c) is to maximise the total value of the portfolio as dictated by the selected options and corresponding project schedule. Eqs.  (A.2d) and (A.2e) enforce that exactly one option from each family is selected and that all projects from the selected options are implemented. Eq.  (A.2f) enforces that all projects start within their allowable window. The budget constraint is given in Eq.  (A.2g), which uses the ancillary variable $z_{pt}$ given in Eq.  (A.2a). Finally, Eqs.  (A.2h) and (A.2i) specify that the variables for option and project selection, respectively, are binary.

## Footnotes

1 This ensured that cyclical precedence constraints could not be generated.

2 Excluding the empty set, which was always taken as the first option in a family.
